# A novel mixed reality system to manage phantom pain in-home: results of a pilot clinical trial

**DOI:** 10.3389/fpain.2023.1183954

**Published:** 2023-06-02

**Authors:** Drupad Annapureddy, Thiru M. Annaswamy, Gargi Raval, Yu-Yen Chung, Balakrishnan Prabhakaran

**Affiliations:** ^1^ The University of Texas Southwestern Medical School, Dallas, TX, United States; ^2^ Physical Medicine & Rehabilitation Service, Veterans Affairs North Texas Health Care System, Dallas, TX, United States; ^3^ Department of Physical Medicine & Rehabilitation, The University of Texas Southwestern Medical Center, Dallas, TX, United States; ^4^ Department of Computer Science, The University of Texas at Dallas, Dallas, TX, United States

**Keywords:** amputation, phantom limb pain, augmented reality, pain, mixed reality (MR)

## Abstract

**Introduction:**

Mirror therapy for phantom limb pain (PLP) is a well-accepted treatment method that allows participants to use a mirror to visually perceive the missing limb. Mixed reality options are now becoming increasingly available, but an in-home virtual mirror therapy option has yet to be adequately investigated.

**Methods:**

We had previously developed a mixed reality system for Managing Phantom Pain (Mr. MAPP) that registers the intact limb and mirrors it onto the amputated limb with the system's visual field, allowing the user to engage with interactive games targeting different large lower limb movements. Feasibility and pilot outcomes of treating patients with lower extremity PLP by using Mr. MAPP at home for 1 month were evaluated in this study. Pain intensity and interference were assessed using the McGill Pain Questionnaire, Brief Pain Inventory, and a daily exercise diary. Function was assessed using the Patient Specific Functional Scale (PSFS). The clinical trial registry number for this study is NCT04529083.

**Results:**

This pilot study showed that it was feasible for patients with PLP to use Mr. MAPP at home. Among pilot clinical outcomes, statistically significant differences were noted in mean current pain intensity [1.75 (SD = 0.46) to 1.125 (SD = 0.35) out of 5, *P *= .011] and PSFS goal scores [4.28 (SD = 2.27) to 6.22 (SD = 2.58) out of 10, *P *= .006], with other outcome measures showing non-significant trends towards improvement.

**Discussion:**

This pilot study revealed that in-home use of Mr. MAPP has potential to provide pain relief and improve function in patients with lower extremity PLP and is feasible. Each scale used provided unique perspective on the functional impact of PLP. Further expanded studies and investigation, including a fully powered clinical trial, with these scales are warranted.

**Clinical Trial Registration:**

https://www.clinicaltrials.gov/ct2/show/NCT04529083, Identifier: NCT04529083.

## Introduction

Phantom limb is a persistent sensation of the missing limb experienced in those who undergo amputations. This is a common event, with up to 90% of those who lose a limb experiencing phantom limb at some point (1). Moreover, between 50% and 85% of those with amputations also experience pain located at the missing limb, a condition termed phantom limb pain (PLP) (2–5). The onset time, severity, frequency, type of pain, stability, and duration of PLP vary, making it extremely difficult to treat (6–8). For some patients, this pain can be intensely debilitating and can detrimentally impact physical, emotional, and mental health, as well as impair function (7). More specifically, activities of daily living (ADLs), sleep quality, mood, mobility, work, and quality of life (QOL) are all commonly negatively impacted (9, 10). Reducing frequency and severity of PLP in these individuals can help restore their function and QOL (11). Currently, the treatments available for PLP include surgical options, non-pharmacologic therapies such as mirror therapy (MT), and pharmacologic intervention (1). However, general consensus is that most current treatment options for PLP show limited evidence of efficacy (1, 12–17).

MT is a commonly used treatment for PLP. Although the existing literature has yet to establish a definitive benefit of MT and further work needs to be done, recent studies suggest that MT may help in alleviating PLP (18–22). The participant places a mirror in a position where it can reflect an image of the unaffected limb, allowing the patient to visually perceive the missing limb (18–20). The patient can then perform different movements or activities with the contralateral limb, which reflects onto the mirror. This image can be interpreted as painless movement of the amputated limb, creating a visual illusion that is thought to generate a positive feedback loop to the motor cortex and may block the pain cycle (18, 19, 23). MT is also theorized to help halt the reorganization of the somatosensory and motor cortex regions, a potential contributory factor of PLP (18, 24). However, MT requires keeping the mirror in a static position and actively maintaining focus on the reflection for a long period of time, both of which make MT cumbersome and ineffective at times (25).

As technologies improved and became more accessible, virtual and mixed reality became incorporated into MT to overcome these problems (26). Different approaches use a head mounted display (HMD) or large screen to project the image of an intact limb in the place of an amputated limb. However, virtual MT comes with its own challenges, the main one being a requirement of many sensors and other equipment that can be difficult to use properly. Additionally, software shortcomings can include unrealistic images, improper limb alignment, or insufficient interaction of the phantom limb with the environment (27, 28).

The Mixed Reality System for Managing Phantom Pain (Mr. MAPP) was developed with the intent to address these shortcomings and allow for use in-home without regular supervision from outside trained personnel (29). Rather than relying on body sensors that need to be correctly placed, Mr. MAPP uses a camera sensor that recognizes the intact limb in real time and superimposes this onto the affected limb within a virtual environment. This virtual environment is concurrently visualized through an HMD. In addition to the lack of body sensors, Mr. MAPP aims to deliver a high level of embodiment through the virtual environmental interaction that the user's phantom limb engages in.

The primary goals of this pilot study are to (1) Investigate the potential clinical utility of and expand upon the feasibility of in-home use of the Mr. MAPP system on pain and function in individuals experiencing lower extremity PLP and (2) Evaluate the utility of different scales in portraying severity and functional impact of PLP and their potential responsiveness to treatment. The insights provided by this study and manuscript will contribute to the understanding of effective PLP treatment and the field of pain management. Moreover, the results of this pilot study will help determine whether expanded investigation into Mr. MAPP's clinical usefulness is justified.

## Methods

### Mr. MAPP system introduction

The Mr. MAPP system creates an immersive experience through the real-time integration of visual data from the intact limb to generate an image of the corresponding missing limb. This is accomplished using a Microsoft Kinect v2 RGB-D sensor, which registers the 3D avatar of the user. A laptop computer runs the software program, and an Oculus Rift HMD displays a virtual setting including an avatar with both limbs intact to the user. As the user repositions their intact limb, the Mr. MAPP system simultaneously adjusts the avatar displayed in the Oculus Rift. Unlike other virtual mirror therapy treatments that exist for PLP, Mr. MAPP does not require any limb sensors. A more detailed account of the Mr. MAPP system design has been previously published (30, 31).

### Clinical study setup

The study was approved by the local institutional review board at the Veterans Affairs North Texas Health Care System (IRB number 18-016). Recruitment for participation occurred at the amputee clinic at this facility in two phases due to interruption as result of the Coronavirus-19 (COVID-19) pandemic restrictions. Phase 1 was conducted pre-COVID (2019–2020), was unfunded, and focused primarily on evaluating feasibility of delivering this intervention at home (31). Phase 2 was conducted after clinical research activities were permitted to resume in 2021 and continued to focus on feasibility and pilot clinical outcomes, which were modified to focus more on pain interference and functional impairments related to PLP as opposed to intensity of PLP.

Health Insurance Portability and Accountability Act (HIPAA) waivers were granted, and all established patients at the clinic with lower limb amputation were screened for inclusion into the study. The inclusion criteria were: Individuals over the age of 18 with a lower limb amputation at least 3 months prior and any reported level or duration of PLP. The exclusion criteria were: Individuals with open wounds or active infection in residual or contralateral limbs; any cardiac or medical conditions that impair their ability to adequately perform exercises; history of seizures; residing further than 60 miles from the medical center; and history of motion sickness attributed to HMDs or other similar immersive virtual environments.

The patients who passed screening were evaluated by a physiatrist for reconfirmation of eligibility, after which they were invited to participate in the study. Those who agreed to participate provided written informed consent to formally enroll in the study.

After completing the consent process, each participant was scheduled for a baseline orientation appointment, where they were provided instructions on how to use the Mr. MAPP system. This approximately 1-h training session occurred in person at the Physical Medicine & Rehabilitation (PM&R) clinic at the Dallas Veterans Affairs Medical Center. A research team member familiarized the participant with the Mr. MAPP system, guiding them through its setup and use. This session also included administration of baseline outcome measure surveys and gave each participant an opportunity to ask any questions they had about the Mr. MAPP system or clarify any instructions. Regardless of familiarity prior to the appointment, by the end of the session, all participants verbally confirmed they had a confident understanding of the system and how to properly use it at home. Because the participant was solely responsible for correct usage of the system at home, extra emphasis was placed on establishing that each participant was comfortable with their responsibilities regarding the study. At the end of the session, each participant was given a complete Mr. MAPP system, including a laptop and camera sensor. In addition, in-home visits to set up the system and reference video demonstrations of system use were available to participants if needed (32, 33). Weekly telephone support and as-needed technical support options were also provided.

The in-home virtual MT protocol involving the Mr. MAPP system included three exercise games, or “exergames”. The participant is instructed to sit at a chair approximately 6 feet away from the Kinect sensor with no objects between the user and system before starting the games. Keeping in mind the limitations of the sensor, a team of physiatrists designed the exergames with the input of physical therapists to focus on large movements that were be able to be performed within the sensor's frame. Each of these was intended to focus on one of the three following large lower extremity movements: knee flexion and extension (Bubble Burst exergame), ankle dorsiflexion and plantarflexion (Pedal exergame), and tandem bilateral lower extremity movement (Piano exergame). The Bubble Burst exergame involved flexing and extending the knee by “popping” bubbles that were slowly ascending (29). The Pedal exergame involved plantarflexing and dorsiflexing the ankle by “stepping” on a pedal when prompted. Lastly, the Piano exergame involved flexion and extension of the hip by “stepping” on specific prompted keys of a virtual piano (30). The exergames were standardized across all participants regardless of severity of PLP to keep the intervention consistent, with the purpose of determining Mr. MAPP's potential effectiveness in alleviating this pain. Each game takes approximately a few minutes to play, with each session lasting approximately 7–10 min total.

Each participant was instructed to complete two sessions of exergames daily for 1 month, during which they filled out a patient diary at the beginning and end of each day's exergame sessions. Treatment length was set to 1 month as it was determined to be an appropriate time for intervention while still considering the limited number of Mr. MAPP systems that could be loaned to participants for the study. Following completion of the 1-month period, the participant was scheduled for a final appointment. Here, the Mr. MAPP system was returned, and the same previous outcome measure surveys were administered again. As well, a user satisfaction survey was also administered to gather data on the Mr. MAPP system's user experience.

The clinical outcome surveys that were administered at the baseline and 1-month appointments in addition to the daily patient diary include the long form McGill Pain Questionnaire (MPQ) and Patient Specific Functional Scale (PSFS). In addition, for the second phase of the study, the Brief Pain Inventory (BPI) was also collected. The MPQ includes three different sections: pain categorization (What does your pain feel like?), impacting factors and change (How does your pain change with time?), and severity of pain (How strong is your pain?) (34). The pain categorization section includes 20 subclasses that are grouped into 4 different pain rating indices (PRIs) for various aspects of pain: sensory, affective, evaluative, and miscellaneous. Furthermore, these 4 PRIs can be summed to a numerical score out of 78 (78 being the maximum score indicating highest total pain) (34). This reliable and validated survey was included in this study with the intention to measure overall levels of pain as well as distinguish the specific modalities of pain that may be more responsive to PLP treatment (35). The PSFS is a valid and reliable scale used to measure activity limitation and loss of function in patients with disabilities (36). Each patient lists activities they feel they are unable to do or have difficulty with, along with their corresponding levels of limitation in these activities (36). The range of the scale is from 0 to 10 for each activity, 0 representing “unable to perform activity” and 10 being “able to perform activity at same level as before injury or problem” (36). In addition to looking at the specific pain types within the MPQ, we included the PSFS in this study to give the participants a chance to directly inform us on what specific activities they had the most difficulty and needed the most help with. The BPI is a reliable and valid scale included as a measure to further assess pain interference in phase 2 (37). The scale runs from 0 (does not interfere at all) to 10 (completely interferes) for 7 interference items (domains of life that pain may interfere with) (37). Lastly, the patient diary includes primarily 2 measures for each day's activities: levels of pain prior to and after treatment and amount of time spent playing the exergames on the Mr. MAPP system. The patient diary is intended to provide insight into daily pain levels through the month and pain immediately prior to and after Mr. MAPP use. All the data was stored on Research Electronic Data Capture (REDCap).

Feasibility measures gathered over the course of the study included proportion of individuals recruited that enrolled in the study and proportion of participants enrolled that completed the study. We also recorded the number of participants who had any level of technical difficulties, what resolving measures were taken, and if the technical issues were resolved. Lastly, at the end of the month, we assessed any adverse effects as result of using the Mr. MAPP system.

The primary statistical analyses that were conducted on the survey data were paired *t*-tests comparing baseline and 1-month values. This included paired *t*-tests for the different subcategories and specific relevant questions within each survey. For the patient diary, paired *t*-tests were conducted comparing pain values before and after daily use of the Mr. MAPP system and between average pain for different weeks. Significance levels were set at *α *= .05 and two-tailed *P*-values were used to determine significance. 95% confidence intervals (CI) for the differences were also determined.

As mentioned above, phase 1 of the study was completed in March 2020 and feasibility and preliminary clinical outcomes of phase 1 was published (31). The main conclusions of the initial feasibility study were that Mr. MAPP was feasible and had potential to improve pain and function in lower limb PLP patients (31). However, we heard anecdotally from participants in phase 1 that pain interference was a more meaningful assessment of impairment than severity of pain as measured by the MPQ and exercise diary. Furthermore, minimal clinical conclusions were able to be drawn due to the limited sample size. The second and final phase of the study began in September 2021 after resuming recruitment to address these findings and complete the pilot study. During phase 2, the BPI was added as an outcome measure to better understand pain interference caused by PLP. This survey was chosen based on recent recommendations of a Veterans Health Administration Work Group (38). The participants from phase 1 and phase 2 were combined to create the dataset for this study. This manuscript more substantially analyzes the preliminary clinical outcomes of participants in both phase 1 and 2, as well as expands upon the feasibility findings in the initial preliminary analysis. Furthermore, we also paid attention to which outcome measures are more meaningful to patients and looked for signals that might suggest responsiveness to PLP treatment.

Throughout the study, adverse event data was monitored and collected, and clinicians were notified and available for any clinical intervention as needed. The clinical trial registry number for this study is NCT04529083. Registration in the database occurred after completion of phase 1 (August 2020) as the previous study primarily focused on feasibility.

## Results

### Feasibility outcome data

**Figure 1:**
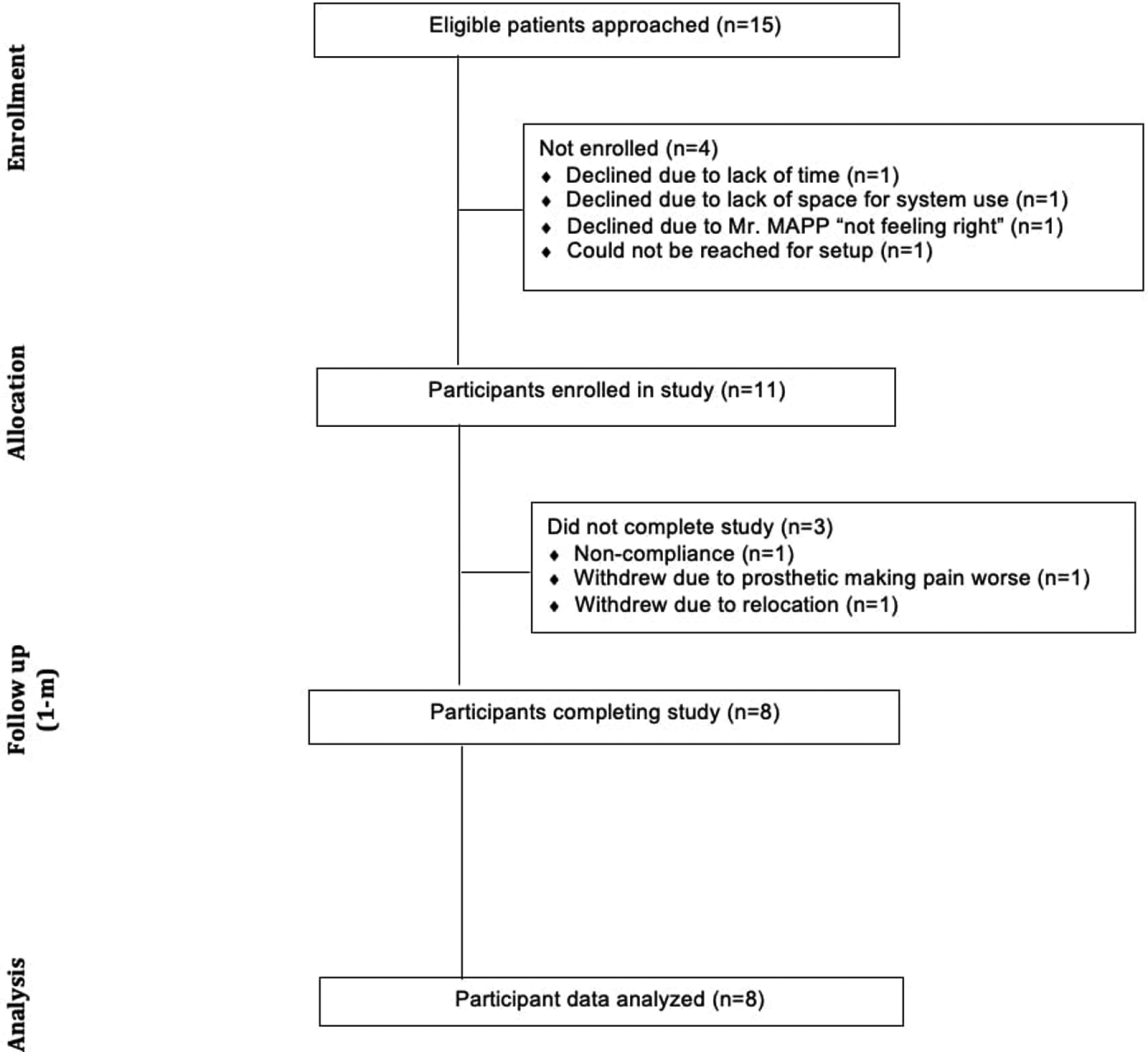
CONSORT diagram showing the flow of participants through pilot clinical trial.

Of the 15 eligible patients approached for recruitment, 11 consented, completed the baseline appointment, and received the Mr. MAPP system to take home (73.3%) (Table 1). The 4 patients recruited that did not eventually enroll were for the following reasons: lack of time, could not be reached for setup, no space for system use, and felt Mr. MAPP “did not feel right”. Of the 11 participants that enrolled, 8 completed the study (72.7%). The progress of participants through the study is illustrated in Figure 1. Regarding demographics, participants who completed the entire study were 100% males, 37.5% of which were African American and 62.5% White. 10 of 11 participants had any level of technical difficulty that required assistance. 3 of these participants utilized phone support, including 1 participant who called for assistance 3 times. The other 7 participants utilized in-person help, including 3 participants who needed another follow-up visit in person. All participants were able to resolve their technical issues following phone support or in-person help. By the end of the study, none of the participants experienced any adverse effects after use of the Mr. MAPP system, and no participants withdrew from the study due to dissatisfaction with Mr. MAPP.

**Table 1:** Participant demographics and level of study involvement.

Participant	Age	Race/Ethnicity	Sex	Type of amputation	Study status
1	60–65	White	Male	Left below knee	Non-compliance
2	50–55	White	Male	Left below knee	Completed
3	70–75	African American	Male	Right above knee	Completed
4	60–65	African American	Male	Right transmetatarsal	Completed
5	60–65	African American	Male	Left below knee	Completed
6	60–65	African American	Female	Left above knee	Withdrew due to prosthetic making pain worse
7	50–55	White	Male	Right above knee	Completed
8	35–40	White	Male	Right below knee	Completed
9	65–70	White	Male	Left below knee	Completed
10	60–65	White	Male	Left below knee	Withdrew due to relocation
11	45–50	White	Male	At hip	Completed

### Clinical outcome data

#### MPQ

Within the MPQ, the overall score decreased by nearly 7 points on an average [38.5 to 31.875 (max score-78)] from baseline to 1 month. While this change was not statistically significant for the overall pain difference, there were significant differences in some specific types of pain. When considering the different subclasses and PRIs within the MPQ, statistically significant improvements were noted in the constrictive pain subclass that decreased from 3.75 to 2 out of 5 (*P* = .049, 95% CI −2.494 to −0.005961) and the evaluative pain subclass/PRI that decreased from 2.125 to 1.625 out of 5 (*P *= .033, 95% CI −0.9469 to −0.05313). While 13 of the other 18 subclasses and all 3 remaining PRIs also saw an average decrease in pain, they did not reach statistical significance. Pain severity level, denoted as the “present pain index” in the MPQ, decreased significantly from 1.75 to 1.125 out of 5 (*P *= .011, 95% CI −1.0577 to −0.1923), demonstrating a 35% average decrease in pain.

#### PSFS

Participant 2 could not come up with any activities/goals when prompted; all other 7 participants provided at least 2 goals. Activity score changes can be interpreted singularly or as an average of all scores (total score) for a user. The PSFS results (Table 2) found that 3 of the 7 participants had total score increases greater than the scale's intrinsic minimum detectable change level of 2 points (90% CI). Participants 5 and 9 had increases in total score from 1 to 3.33 and 6 to 8.67, respectively. The largest difference was recorded in participant 11, whose total score increased from 2 to 10 (complete recovery in ability to perform activity). Considering activities by themselves, 5 activities had individual score increases that were greater than the scale's intrinsic minimum detectable change level of 3 points (90% CI): participant 5's activity 2 (1 to 4), participant 9's activity 1 (5 to 8) and 2 (7 to 10), and participant 11's activity 1 (0 to 10) and 2 (4 to 10). Furthermore, when considering the summation of all activity scores across all participants, a paired *t*-test found that the average individual activity score increased significantly from 4.28 to 6.22 (*P *= .006, 95% CI 0.6291 to 3.2598) from baseline to 1 month (45% average increase). Average total score also increased from 4.31 to 6.36, but this value was not found to be statistically significant.

**Table 2:** Patient specific functional scale goals and baseline and 1-month data.

Patient specific functional scale (PSFS) data							
Participant	Visit	Activity 1	Activity 1 Score	Activity 2	Activity 2 Score	Activity 3	Activity 3 Score
3	Baseline	Cooking	7	Cleaning House	6	Walking Without Crutches	6
	1-Month		8		7		8
4	Baseline	Playing With Grandkids	6	Getting In and Out of Shower	6		N/A
	1-Month		7		6		N/A
5	Baseline	Walking	1	Standing for Long Periods	1	Sleeping (Staying Asleep)	1
	1-Month		3		4		3
7	Baseline	Participate in Recreational Activities	5	Fall Asleep Easier	6		N/A
	1-Month		6		4		N/A
8	Baseline	Long Walk (>300 Meters)	3	Prolonged Standing	3	Daily Work/Household Activities	4
	1-Month		3		3		4
9	Baseline	Falling Asleep	5	Staying Asleep	7	Interacting With People	6
	1-Month		8		10		8
11	Baseline	Falling and Staying Asleep	0	Concentrating on Tasks	4		N/A
	1-Month		10		10		N/A

Range: 0–10. 0 = unable to perform activity, 10 = able to perform activity at same level as before injury or problem.

#### BPI

The BPI survey data was only gathered in phase 2 of this study [the last 4 participants who completed the study (participants 7, 8, 9, 11)]. Average interference across all 7 interference items for each participant decreased from 4.75 to 1.39 out of 10 (*P *= .037, 95% CI −6.3181 to −0.3962). When examining the specific interference items, significant improvement in interference were noted in 4: general activity (4 to 0.75, *P *= .014, 95% CI −5.2522 to −1.2478), mood (4.75 to 2, *P *= .048, 95% CI −5.4675 to −0.03247), relations with other people (4.25 to 1, *P *= .023, 95% CI −5.6368 to −0.8632), and enjoyment of life (4 to 1.25, *P *= .035, 95% CI −5.1368 to −0.3632). All 3 other interference items (walking ability, normal work, and sleep) had average decreases in scores that did not reach statistical significance. Measures of pain intensity in the last 24 h (out of a scale from 0 to 10) found an average decrease in worst pain (5.74 to 4.25), least pain (2.5 to 1.5), average pain (3.75 to 3.5), and pain right now (3.5 to 2). However, none of these values reached statistical significance.

#### Exercise diary

**Figure 2:**
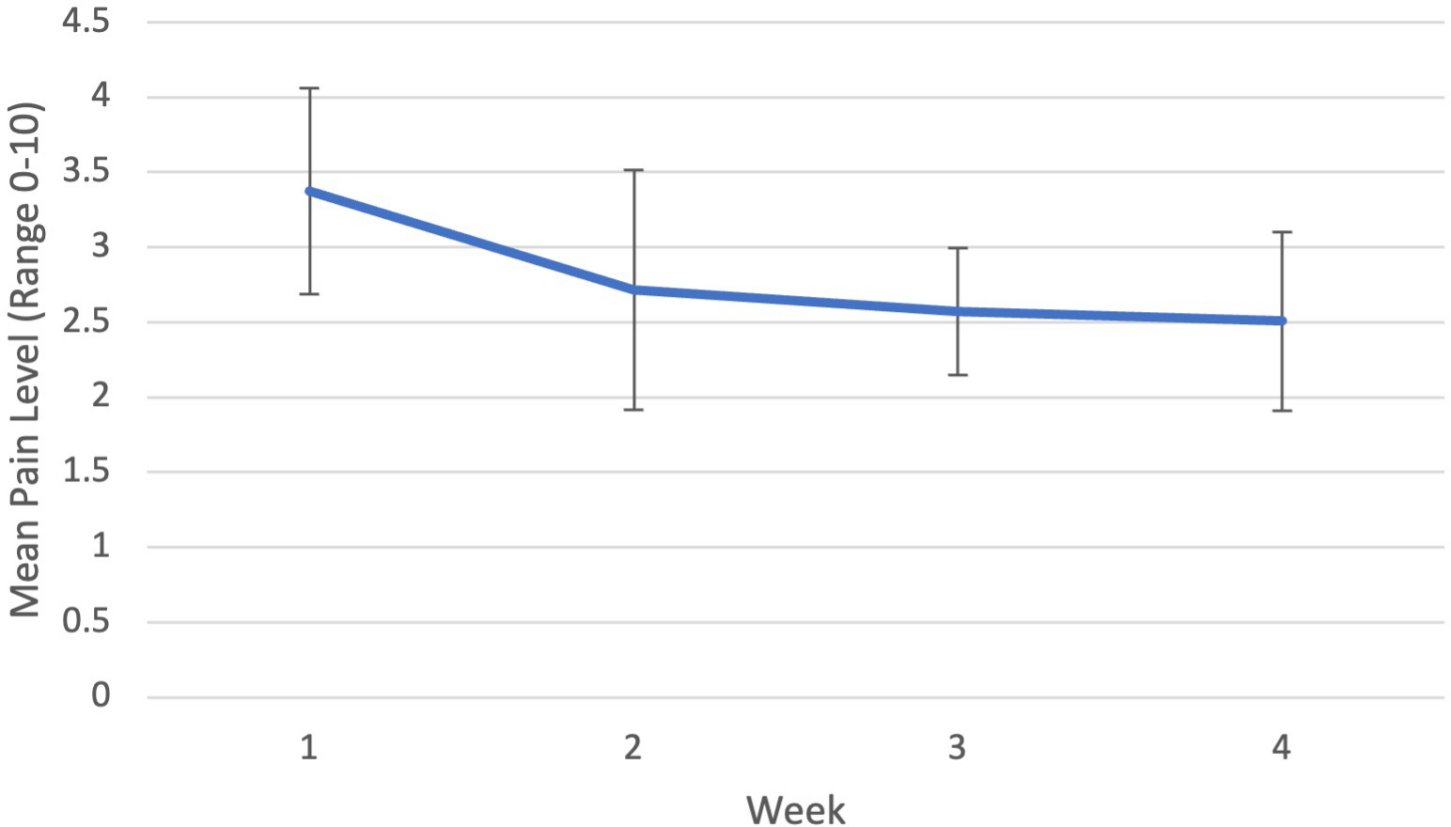
Patient diary average weekly pre-exercise pain levels.

A paired *t*-test comparing pain immediately before and after use of the Mr. MAPP system found a statistically significant modest decrease in pain from 2.92 to 2.71 out of 10 (*P *= .0013, 95% CI −0.3378 to −0.08424). Only 4 of 8 participants who completed the study (participants 2, 5, 7, 9) filled the patient diary out consistently, for all 4 weeks. For these 4 participants, an average weekly level of pain for each day before use of the Mr. MAPP system was calculated (Figure 2). There was a significant difference in pre-exercise pain (range: 0 to 10) between week 1 and 3 (3.375 to 2.571, *P *= .013, 95% CI −1.2842 to −0.323). There was also a significant decrease between week 1 and 4 (3.375 to 2.51, *P *= .019, 95% CI −1.4645 to −0.2736), demonstrating a 25% average decrease in pain.

### User survey

One relevant question on the patient satisfaction survey asked if any participant used traditional mirror therapy in the past. Only participant 8 stated that he had used MT before, and when prompted to compare that experience to the Mr. MAPP system, he believed that mixed reality-based MT was more realistic than the traditional MT.

## Discussion

The findings from our study indicate that use of Mr. MAPP in-home to assist with symptom management of phantom limb pain is feasible. A majority of those recruited enrolled the study, and a similar majority of those enrolled completed the study. All participants were able to resolve any technical difficulties over the phone or after in-person visits. Furthermore, there were no adverse effects as result of Mr. MAPP use, and no participants withdrew due to dissatisfaction with the system. These findings support the conclusions of the phase 1 feasibility study and suggest that further studies, including large scale randomized clinical trials, for the Mr. MAPP system are feasible and realistic (31).

Several pertinent findings from this study are related to the utility of the survey tools in measuring the pain intensity and function of those experiencing PLP. While the MPQ and exercise diary measured the level of pain severity directly, the BPI measured interference in daily living, which may be a more relevant indicator of quality of life as impacted by pain. Pain itself is an important consideration when determining effective treatment modalities, but the ultimate goal of medical therapy is typically to improve daily living functioning. Meanwhile, the PSFS measured the ability of the participants to perform various self-identified activities. The requirement of the participants to determine these activities highlights the aspects of their lives that were most important to them and thus most relevant in measuring treatment effectiveness. In addition, the PSFS allows clinicians and researchers to custom address the unique functional limitations that PLP poses to each patient. Providing a treatment option that can produce significant functional improvement in a self-identified task is more meaningful to a patient compared to pre-selected functional tasks that may not be relevant to them. Along with the MPQ and exercise diary, the BPI and PSFS are both useful tools that should be utilized in further investigation of mixed reality PLP treatment.

A preliminary analysis of outcome instruments and their different components yielded interesting information regarding how Mr. MAPP may affect pain and function in patients with lower limb loss and PLP. The MPQ data analysis revealed that specific types of pain−constrictive and evaluative−improved with 1-month in-home use of Mr. MAPP. This is similar to prior research suggesting that specific types of pain characteristics (motor or movement types of pain) are more responsive to treatment with the motor-imagery based treatment approach (e.g., mirror therapy, Mr. MAPP etc.) (39, 40). More investigation is needed to conclusively determine the effects of Mr. MAPP on these and other categories of pain.

Analysis of overall pain intensity change revealed mixed results. In the MPQ, an indicator of severity of pain is the “current pain level”. This showed a statistically significant decrease from baseline to 1 month, although clinical significance is uncertain. It is unclear if average pain intensity is a reliable indicator for evaluating effect on pain in patients with PLP because of the reported high inherent variability in pain intensity (41).

Compared to average pain intensity, analysis of immediate post-treatment pain intensity change provided meaningful information. The patient diary provided useful information regarding progress in immediate post-treatment pain relief. Paired *t*-tests comparing before and after use of the Mr. MAPP system each day found a modest decrease in pain. Future comparison of immediate post-treatment pain relief with Mr. MAPP to an appropriate control group could help evaluate this further. The patient diary also allowed for analysis of weekly pain intensity change. Paired *t*-tests between different weeks indicated a significant difference in pre-exercise pain levels between week 1 and weeks 3 and 4 suggesting that baseline pain levels decreased incrementally during the treatment period. This further supports the conclusion that Mr. MAPP system could help decrease the severity of pain. Results from previous literature on the effectiveness of other virtual MT therapies similarly correspond with the decreases in pain found in this study (26). Furthermore, with supplemental data from the patient diary, we were able to elucidate more comprehensive and specific findings than with just baseline and 1-month data. While the findings may not be clinically significant, the most important finding from implementation of the exercise diary is its utility in providing a large number of datapoints immediately prior to and after in-home mixed reality mirror therapy, helping minimize confounding factors on levels of pain.

As previously noted, interference from pain may be more meaningful to patients and clinicians compared to pain intensity. Adding the BPI in phase 2 of the study allowed for further examination into how each participant's pain affects their overall general emotional, interpersonal, and physical functioning. While only half of the study population were able to complete this survey, the findings were useful, nonetheless. This study found that the Mr. MAPP system possibly decreased overall pain-related interference and specifically that of general activity, mood, relations with other people, and enjoyment of life. These are all considered daily life activities by the BPI, and this data points to Mr. MAPP's potential ability to increase overall functioning by decreasing pain interference.

The PSFS focused on the function to perform various activities with respect to their pain. In these activities, we saw a marked increase in general ability to perform activities in at least 3 of the 8 patients. The most noticeable patient to see these results was patient 11. He was able to return from scores of 0 (not being able to perform activity at all) and 4 to both 10's (being able to perform the activity as well as before his amputation). After 1 month, he was able to fully perform his listed activities that he previously couldn't do well, if at all. For individual activities, there was a significant increase in scores overall across the board. An important goal of this study was to evaluate the interaction of pain and function. Higher scores indicate better ability to perform these activities and higher overall physical function, possibly as result of pain loss. These findings suggest the potential effectiveness of Mr. MAPP in decreasing the pain-related interference of physical functioning. More importantly, the PSFS provided invaluable insight into how to measure the effectiveness of a potential treatment for lower extremity PLP.

The Mr. MAPP system provides a feasible, novel method to implement treatment in the comfort of a patient's own home without healthcare provider supervision. This low-cost solution is a step towards providing equitable, safe, and realistic therapy for those who experience any varying degrees of PLP. Along with pointing to the future utility of the scales used, the findings from this study provide notable support for serious future consideration of the Mr. MAPP system as a tool for pain relief and to increase function. Multiple scales and methods of measurements suggest Mr. MAPP could help reduce severity of pain, alleviate certain types of pain, increase physical function in patient-specific activities, and/or improve overall daily functioning.

## Limitations

While statistical significance was assessed in this study, the small sample size of this clinical pilot study limits the generalizability of outcome changes observed. However, these findings do suggest that Mr. MAPP system is a potentially useful therapeutic tool in alleviating pain and improving function, meriting further investigation. A fully powered clinical trial comparing Mr. MAPP with a MT or other standard-of-care (such as pharmacological or physical therapy) control group could help definitively quantify the effects of Mr. MAPP on these measures. Additionally, there were several variables that were not controlled for in the study. These include the etiology and level of the amputation, severity and duration of PLP, prior exposure to traditional MT, and number of minutes spent using the Mr. MAPP system. Lastly, analgesic use prior to and during the month course of system use was not assessed or controlled for. Any observed decreases in pain after Mr. MAPP use could be attributed to unmeasured changes in analgesic use. However, this pilot study provided meaningful information and insight into a novel treatment procedure for lower extremity PLP and the accompanying scales used. With additional follow-up confirmatory studies, Mr. MAPP has potential to be a reasonable alternative in-home treatment option for those who experience this pain and its subsequent interference in their lives.

## Conclusions

The results of this clinical pilot study suggest that Mr. MAPP, a novel, mixed reality based in-home treatment system, is feasible and has potential to decrease pain intensity and interference and improve function in patients with lower extremity PLP. The surveys and scales used, including the BPI, PSFS, exercise diary, and MPQ, each provided a unique perspective on the severity of PLP and associated impairment. Further investigation with a fully powered clinical trial, including an appropriate control group and adequate sample size, could help establish a novel in-home virtual mirror therapy as a viable future treatment option for limb loss and phantom limb pain.

## Data Availability

The raw data supporting the conclusions of this article will be made available by the authors, without undue reservation.
